# Computer vision techniques for high-speed atomic force microscopy of DNA molecules

**DOI:** 10.1088/1361-6528/ade888

**Published:** 2025-07-16

**Authors:** Nicholas Driver, Andrey Mikheikin, Sean Koebley, Morteza Mostashari, Loren Picco, Seth I Berger, Jason Reed

**Affiliations:** 1 Department of Physics, Virginia Commonwealth University, 701 W Grace St, Richmond, VA, United States of America; 2 H. H. Wills Physics Laboratory, Tyndall Avenue Bristol, Bristol, BS8 1TL, United Kingdom; 3 Children’s National Medical Center, Center for Genetic Medicine Research, Rare Disease Institute/Division of Genetics and Metabolism, Washington, DC, United States of America; 4 Massey Comprehensive Cancer Center, Virginia Commonwealth University, Richmond, VA, United States of America; 5 Virginia Institute for Engineering and Medicine, Virginia Commonwealth University, Richmond, VA, United States of America

**Keywords:** atomic force microscopy, neural network, inherited disease, DNA, CRISPR

## Abstract

High-speed atomic force microscopy (HSAFM) can produce thousands of topographic, nanoscale images over a small area. One emerging application of this technique is the detection and sizing of single DNA molecules derived from biological experiments and genetic testing. Using HSAFM images, researchers can visually categorize healthy and mutant DNA based on size and sequence-specific labeling, leading to rapid, high precision diagnostics. However, manually sifting through large numbers of images is time consuming and labor intensive, presenting a bottleneck for sample analysis. In this paper we look at how deep learning methods like object detection and image classification can assist in streamlining the process. To instantly assess image quality on data collected from trinucleotide repeat expansion disease samples, a fully convolutional network (FCN) was compared against Laplacian of Gaussian and fast Fourier transform methods. The FCN performed the best, reproducing human categorizations with an accuracy of 96% and an AUC of 0.990. Additionally, using the YOLOv8 architecture, we have developed an object detection model capable of detecting marked DNA from patients with Fragile X syndrome with an average precision of 0.966. The object detection model searched through 20 000 images containing DNA molecules and identified 248 marked molecules, of which 33 were real targets, greatly reducing the time taken to find target molecules for diagnostics. The integration of machine learning techniques in HSAFM systems shows promise to enhance the data collection and analysis process for genomics-based disease diagnosis.

## 1. Introduction

High speed atomic force microscopes (HSAFMs), operating in both contact and tapping mode, have seen extensive use in nanoscale surface imaging since their introduction and subsequent development over the last twenty years [1–5]. Their increased frame rates have enabled the study of real-time processes in ambient air, as well as liquid environments. Contact mode HSAFM is particularly effective for those samples where increased lateral forces are not a concern, because it can achieve high pixel rates per second without requiring rapid active force control. Example materials imageable with contact-mode HSAFM include many metal-based samples like metallic nanoparticles, 2D materials, such as graphene and phosphorene nanoribbons [6], and various sizes of DNA molecules [7, 8]. HSAFM is very well suited for single molecule counting experiments, as a large area can be covered in a small amount of time, allowing the buildup of statistics via measurement of numerous individual molecules.

Recent work from Mikheikin *et al* demonstrated the applicability of this technology in molecular diagnostics, using HSAFM to distinguish between mutant and non-mutant DNA molecules on the basis of their physical length, achieving an ROC-AUC score of 0.988 [8]. ROC-AUC stands for receiver operating characteristic curve—area under the curve, and is the area under a plot of the true positive rate vs the false positive rate at many different classification thresholds. The area under this curve measures a statistical model’s capability to separate positive and negative classes in binary classification (1.0 is a perfect score, while 0.5 means the model is as good as flipping a coin). The molecules were generated using PCR-amplified samples from patients with a form of acute myeloid leukemia that is caused by an insertion polymorphism in the FLT3 gene. This method does not require a large amount of sample genetic material and is more sensitive than the current field standard test based on electrophoresis. Additionally, very long DNA segments can be imaged with HSAFM and when paired with the use of sequence-directed labels, as in Paulson [9], target regions can be distinguished from one another. This work suggests that HSAFM may particularly be useful for diagnostics of repeat expansion diseases, a class of disease primarily affecting the nervous system, in which a repeat mutation of certain nucleotides in the genome occurs. Fragile X syndrome (FXS) and Huntington’s disease are examples of diseases caused by such mutations. Depending on the size of the mutation, repeat expansion diseases can manifest with markedly varied phenotypes [9]. In the case of FXS, an X-linked disease affecting the FMR1 gene, affected individuals have a number of CGG repeats causing protein misfolding, silencing FMR1 expression. Unaffected individuals have 5–44 CGG repeats, individuals with intermediate expansion have 45–54 CGG repeats, individuals with premutation expansion have 55–200 repeats, and those with a full mutation have greater than 200 repeats [10]. With the FMR1 gene being on the X chromosome, females in the population can be carriers of FXS due to X chromosome inactivation. FXS with full allele mutation occurs in about 1 in 7000 males and 1 in 11 000 females, though asymptomatic female carriers are estimated to be 1 in 180–250 [10]. Female carriers of premutations are at increased risk of adult onset ataxia and premature ovarian insufficiency. Due to the high-resolution images obtained from the HSAFM system, 1–2 nm per pixel, these diseases can not only be diagnosed, but the physical extent of the repeat expansion can be characterized with an accuracy of ∼5 base pairs. Since the mutation can be passed down through generations, this potential for rapid screening of asymptomatic individuals is very relevant to people looking to have children.

This HSAFM sizing of DNA, along with its development and testing, presents unique challenges, however. The collection of thousands of images is a laborious task, even with the benefit of high-speed scanning. In contrast with the high-speed tapping mode designs of the groups of Ando and Hansma [4, 5], the contact HSAFM used in [7] does not use active feedback to manage cantilever tip-surface force. Additionally, the presence of other nanoscale surface contaminants, changes in cantilever tip quality, and environmental vibrational noise can drastically reduce image quality during data collection. In practice, the contact mode HSAFM operator is often required to manually optimize collected data, by visually inspecting images as they are collected in real time and adjusting instrument parameters during scanning. Furthermore, target molecules may not be present in every frame, and in general might have areas of higher and lower concentration across the entirety of the surface, creating yet another real-time challenge for operators, who must maximize scanning in areas of high target molecule concentration (and low contamination). Target molecule concentration can in general be very low, thus considerable time and effort are spent on post-processing data to maximize the number of diagnostically usable analyte molecules.

Here we present image processing-based solutions to two major challenges: First, we present a fully convolutional network (FCN) capable of inferring image quality directly from input images in real time. The algorithm effectively replaces the HSAFM operators’ decision-making during data collection, producing a number between 0 and 1, where higher numbers correspond to ‘good’ (spatially sharp, low noise, high contrast) images and lower numbers correspond to ‘bad’ (noise, blurring, low contrast) images. The time required for processing a single image and sending it through the FCN algorithm is on the order of milliseconds, while our typical time to collect one full frame image is between 0.5–1 s, leaving ample time for processing, decision making, and slight adjustment of cantilever height. Even without full automation of the instrument itself, this FCN algorithm is useful in that it serves to filter out lower quality images, which are less useful in the disease diagnosis process due to potentially inaccurate apparent molecule size.

Second, we present an object detection network, based on the recently released YOLOv8 architecture [11, 12], which can autonomously search through images to find molecules of interest. This algorithm was trained on manually labeled data from an ongoing study involving the measurement of nucleotide repeat expansions, and achieved a high average precision (AP) score of 0.966, meaning that it accurately classified and localized target molecules within images. The use of this algorithm for post-processing greatly reduces time taken to parse through data while simultaneously removing the propensity for false negative results, outperforming human operators by finding target mutated molecules that were missed during manual inspection. In the case of detecting single-copy length polymorphisms directly from a preparation of human genomic DNA, target molecules will be present at less than 1 in 10 000 DNA strands. This algorithm can thus be thought of as a method to filter through unwanted molecules in a ‘needle-in-the-haystack’ style of problem.

## 2. Materials and methods

### 2.1. Image quality detection

**FCN, LOG, and FFT algorithms, and training data.** In general, to create a measure of microscope image quality, images need to be categorized as ‘good’ or ‘bad’ i.e. blurry or sharp, and deep learning is not the only tool available to achieve this goal. In addition to the FCN, additional image processing techniques for detecting blurred images, not based on deep learning, were chosen to provide comparative analysis. Two commonly used methods of detecting blurred images in CCD/digital camera applications are the Laplacian of Gaussian (LOG), and a high-pass filter using the 2D Fourier Transform of the image [13–19]. Blurriness was specifically chosen in this situation to be the largest contributing factor to overall usability of images, because both incorrect cantilever pressure and dullness of cantilever tip create situations where the accuracy of measured DNA strand length is reduced. These situations are very commonly encountered during data collection on the HSAFM system and require the aforementioned manual intervention of AFM operators.

The LOG method is an edge detection filter, with a preprocessing step of Gaussian blurring to reduce sensitivity to noise. Blurrier images will have less defined edges, and so the variance of the filtered image will be lower for blurry images and higher for focused images. To make a binary classification, we choose a variance threshold, above which images are considered ‘good.’ The fast Fourier transform (FFT) based blur detection, on the other hand, works like a high pass filter. Sharper images will have more high frequency content, and so after filtering, the average pixel brightness of a blurry image will be lower than that of a focused image. For classification in this case, we choose a mean intensity threshold, i.e. for each image the mean intensity of the entire image after filtering is calculated. Both the LOG and FFT methods were implemented as custom code and optimized for our application, using Python and OpenCV.

**Figure 1.**
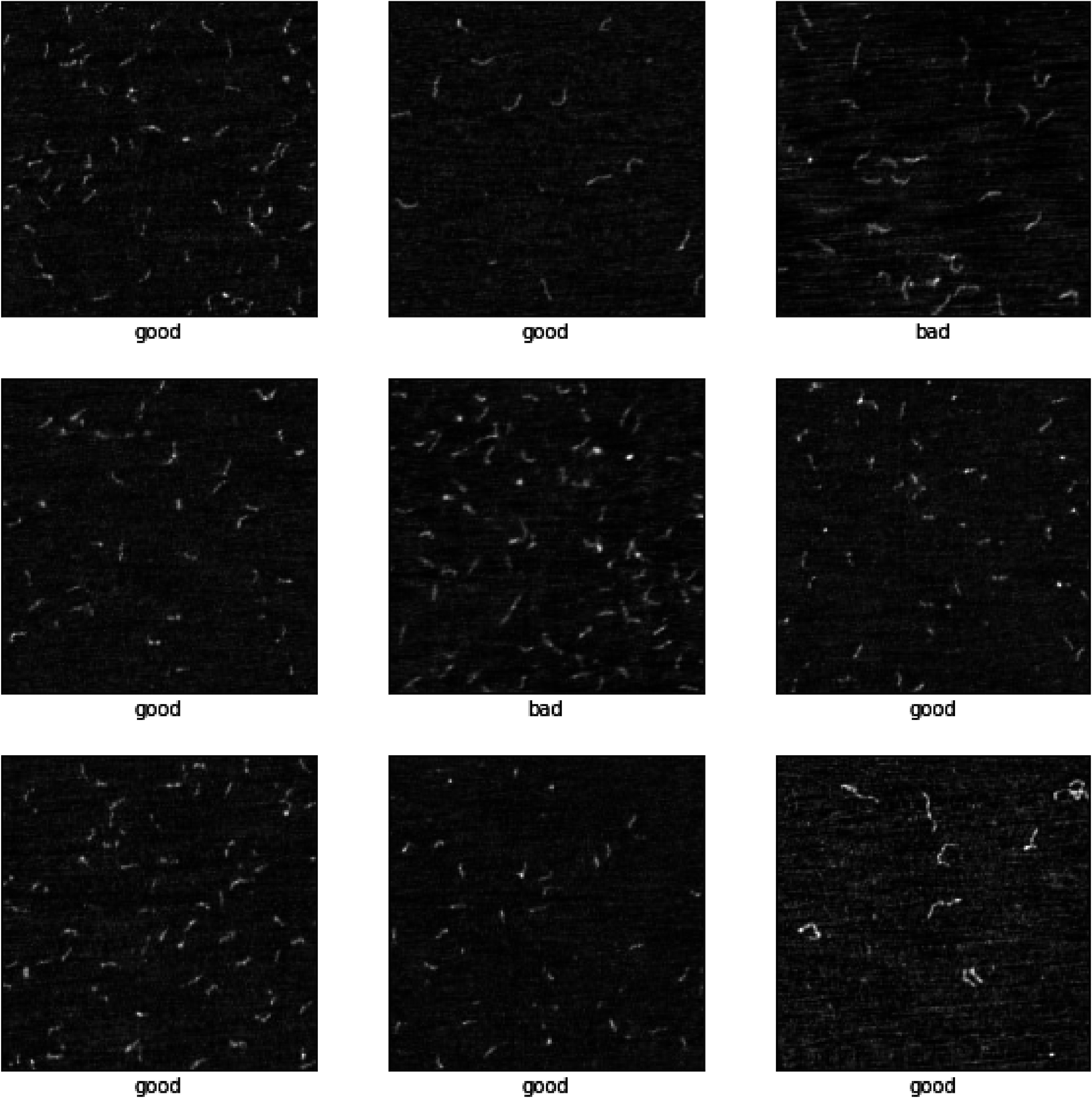
Labeled HSAFM images for the FCN algorithm, sorted into two classes: ‘good’ and ‘bad’. Good images represent acceptable cantilever pressure, while bad images represent cantilever pressure that is too high. Scale: image size is 2 × 2 *μ*m.

All image data was collected using a custom HSAFM described previously [7]. To design, test, and verify the image quality and object detection algorithms, ground-truth labeled datasets were created. Data for the FCN consisted of HSAFM images of 100–300 bp long PCR products, and was collected as part of an ongoing study on repeat expansion mutations [20], and later categorized by three trained AFM operators, with each of the three resulting labeled datasets being kept separate—hereafter referred to as Dataset A, Dataset B, and Dataset C. Dataset C was kept out of the training and validation process, and used as a testing or ‘holdout’ dataset. Prior to training and inference, all images were rescaled to the same height range, then processed to normalize, improve contrast, and remove high features like dust and other nanoscale contaminants. Examples of ground truth labeled images can be found in figure 1. This manual labeling process resulted in 1300 training and validation images categorized as either ‘good’ or ‘bad’ (Dataset A around 900 and Dataset B around 400), with the testing Dataset C being 100 images, for a grand total of 1400 images. This FCN was a custom designed architecture, using convolutional layers, batch renormalization [21], max pooling, and global average pooling, as outlined by Lin *et al* [22]. Using max pooling layers between each convolutional layer has been shown to make networks more invariant to spatial translations of image data, speed up convergence, and additionally reduce overall network size [23, 24]. Choosing to omit fully connected layers, opting for a FCN, kept the total number of parameters for the network to around 520 000, making it easier to optimize hyperparameters on an Nvidia RTX 3070 GPU. All layers used rectified linear unit activation functions, except for the final sigmoid output layer which maps the output to a number between 0 and 1. As this is a binary classification algorithm, standard binary cross-entropy was chosen as the loss function, and training was done using the ADAM optimization algorithm built in to the Tensorflow python library [25]. The loss function is, broadly, a mathematical measure of how correct or incorrect the network’s guess is for a particular iteration, and is used during the gradient descent-based training process. It can take different forms, and does have an indirect effect on the performance of the model.

As previously mentioned, data for the FCN was manually labeled by three different AFM operators and kept separate. This provided an opportunity to explore how the network responded to training and testing on different combinations of the datasets. For example, Training on Dataset A and testing with Dataset B might produce different results than vice versa, since the neural network would have the bias of the operator who labeled Dataset A trained into it. Different HSAFM users might have different opinions about what constitutes a ‘good’ image, perhaps one operator tends to be more cautious than the other. The best-case scenario, with the greatest ability to generalize to test data, might then be a training set that incorporates the labeled data of several operators, combining their opinions together and promoting the best diversity in the training set.

### 2.2. Object detection

**Nanoparticle patterning to identify molecules containing the FMR1 locus.** FXS affected samples viewed on the HSAFM system can also be differentiated using protein markers, as a gRNA-Cas9 complex can be customized to selectively bind to sites on the FMR1 gene. Cas9 here serves two purposes, first is to specifically mark the FMR1 gene on the images and second is to provide a physical barrier to aid in the DNA purification process. Regarding the former, DNA has a diameter of approximately 2 nm, and Cas9 has a diameter of 10 nm, and since the HSAFM obtains topographical data, the difference between the heights of the objects is clear in the images taken. Regarding the latter, this study does not use PCR amplification due to the high GC content of FMR1, but instead uses the CaBagE negative enrichment described by Wallace *et al* [26]. The CaBagE method positions the sequence of interest between two attached Cas9 proteins and removes the rest of the genetic material using exonucleases. The Cas9 proteins act as a physical barrier, stopping the exonucleases from digesting the FMR1 gene allowing for specified analysis. The gRNA-Cas9 complexes attach to three sites on the FMR1 gene, two at either end of the repeating region and one as a control. Since pixel distance is known, the distance between these protein markers can be calculated, to determine if samples are positive or negative for FXS and other repetitive genetic disorders.

**Synthetic DNA samples.** Synthetic DNA used for training the FMR1 detection network was purchased from Integrated DNA Technologies (Coralville, IA). Locations of gRNA are colored green and PAM sites are in red:

CTCTCATGTGTGACAGTTCAACAGCGTTGATCACGTGACGTGGTCATCGATAAGCTTTAATGCGGTAGCCGGGGG TTCGGCCTCAGTCAGGTTTATCACAGTTAATCGGTTTCACTTCCGGTGGAGGGATTGCTAACGCAGTCAGGCACCG TGTATGAAATCTAACAATGCGCTCATCGTCATCCTCGGCACCGTCACCCTGGATGCTGTAGGCATAGGCTTGGTTAT GCCGGTACTGCCGGGCCTCTTGCGGGATATCGTCCATTCCGACAGCATCGCCAGTCACTATGGCGTGCTGCTAGCGC TATATGCGTTGCCGGCGCTAGCAGGGCTGAAGAGATGCATTTCTATGCGCACCCGTTCTCGGAGCACTGTCCGACCGC TTTGGCCGCCGCCCAGTCCTGCTCGCTTCGCTACTTGGAGCCACTATCGACTACGCGATCATGGCGACCACACCCGT CCTGTGGATCCTCTACGCCGGACGCATCGTGGCCGGCATCACCGGCGCCACAGGTGCGGTTGCTGGCGCCTATATCGCC GACATCACCGATGGGGAAGATCGGGCTCGCCACTTCGGGCTCATGAGCGCTTGTTTCGGCGTGGGTATGGTGGCAGG CCCCGTGGCCGGGGGACTGTTGGGCGCCATCTCCTTGCATGCACCATTCCTTGCGGCGGCGGTGCTCAACGGCCTC AACCTACTACTGGGCTGCTTCCTAATGCAGGAGTCGCATAAGGGAGAGCGTCTCCCCCTTTCCTAAACATCATCT CCCCCAGGGATCCGGGCCTGTCGTGTGGGTAGTTGTGGAGGAGTCAGCTTCTTACGGTG.

The overall length of the synthetic DNA, and the relative spacing of the gRNA sites corresponds to the expected length of the consensus wild-type FMR1 fragment that would be enriched using the CaBagE described below. In the corresponding real FMR1 fragments, the repeats would occur between the first and second gRNA sites. The repeats themselves cannot be directly synthesized due to GC content, so sequences in the synthetic fragment between markers were randomized while preserving marker spacing. Anonymized wild-type female genomic DNA purchased from Promega was used as input material for the enrichment reaction.

**CaBagE FXS enrichment and Cas9 labeling.** Five micrograms of wild-type female genomic DNA (Promega) were treated with 10 U of HpyCH4V and 20 U of Mung Bean nuclease (both enzymes from New England Biolabs) in 50 *µ*l of 1× CutSmart buffer (New England Biolabs) for 1 h at 37 C followed by purification with MinElute purification kit (Qiagen). crRNA and tracRNA were obtained from Integrated DNA Technologies and gRNA were prepared according to protocol from the manufacturer (for sequences Guide RNA sequences: gRNA1-CCTGACTGAGGCCGAACCCC; gRNA2-CTCTTCAGCCCTGCTAGCGC; gRNA3-TCCACAACTACCCACACGAC). Labeling with gRNA-Cas9 was conducted in 20 *µ*l of 20 mM HEPES, 100 mM NaCl, 0.1 mM EDTA buffer (pH = 6.5) with 0.2 *µ*M Cas9 (New England Biolabs) and 0.8 *µ*M of each gRNA for 1 h at 37 C followed by addition of 0.5 *µ*l of 5% glutaraldehyde, incubation for 20 min at room temperature, addition of 7 *µ*l of 3 M TRIS (pH = 8.0), incubation for 20 min at room temperature and dialysis with 0.1 *μ*m VCWP membrane (Millipore Sigma) against 45 ml of MQ water for 45 min. Exonuclease cleavage reaction was conducted according to [26] in 50 *µ*l of 1× NEBuffer 4 (New England Biolabs) with 40 U of Exonuclease I, 100 U of Exonuclease III (both enzymes from ThermoFisher), 20 U Lambda Exonuclease (New England Biolabs) overnight at 37C followed by AMPure (Beckman Coulter) purification with 0.8:1 beads: solution ratio. Sample was deposited on freshly cleave mica in 5 mM TRIS (pH = 8.0) and 5 mM magnesium chloride solution, incubated for 5 min at room temperatures, washed 3 times with 200 *µ*l of MQ water and baked for 10 min at 120C.

**Figure 2.**
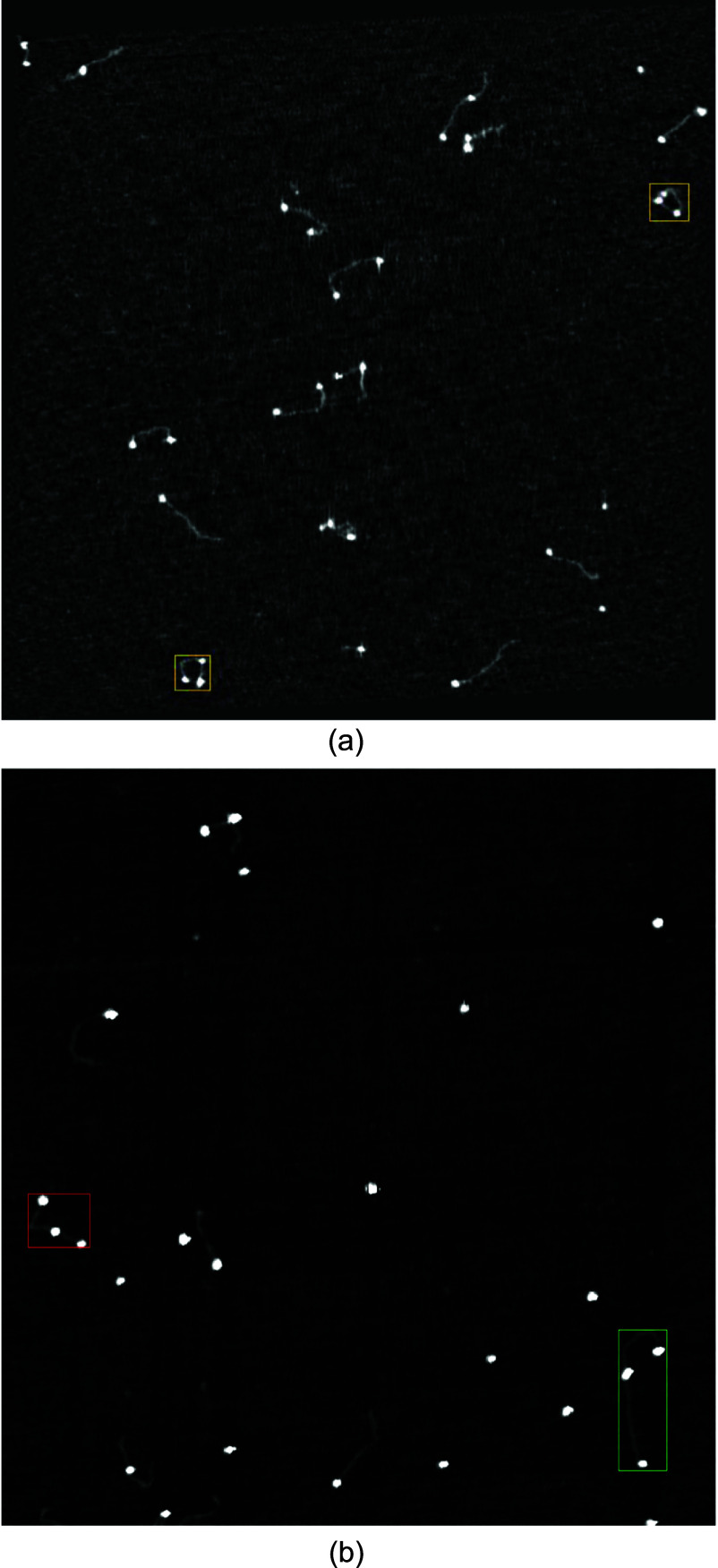
(a) Example image from the dataset for the object detection network. DNA has CRISPR markers (bright white circles) attached. Only the strands with exactly three labels are the target molecules, outlined in yellow for this image. (b): An example image from the training dataset for the object detection network, featuring one instance of the ‘triple labeled’ class (red bounding box), and one instance of the ‘triple labeled long’ class (green bounding box).

**Object detection network and training data.** Data for the object detection network was collected as part of an ongoing study on FXS, characterizing the number of CGG repeats in the FMR1 gene by direct length measurement. Images were again labeled manually with a data labeling program, whose outputs were organized into the desired input format for YOLOv8. In this study, molecules corresponding to the FMR1 gene were marked with Cas-9 enzyme markers, so that any molecule corresponding to the correct DNA segment will have the correct pattern of labels (figure 2(a)). Bounding boxes were drawn around any molecule with exactly three markers, with box coordinates being saved as metadata. The object detection algorithm’s task, then, is to find only those strands that have exactly three labels. Images containing these strands can then be fed to already existing image processing schemes for measuring the molecule length.

A major obstacle in the creation of this training dataset is the fact that human DNA in the FMR1 repeat region cannot be amplified with PCR due to high CG content. It was decided to use synthetic DNA, along with other chemical enrichment methods, described earlier. Due to its inherent purity, synthetic DNA is more often perfectly marked by Cas-9 markers, leading to far greater than 1/10 000 target molecule ratio expected in real samples. Using synthetic DNA for training drastically sped up the process of generating ground-truth labeled data, while human cell line samples were later prepared for the purpose of testing. This testing data was used to evaluate the network’s performance when target molecule concentration and purity of samples were much lower.

The manual labeling process for this algorithm resulted in around 1400 images, with 1530 instances of the ‘triple labeled’ class. 100 images in the dataset were background images, containing no examples of the target molecules but potentially containing other DNA strands and stray markers/contaminants. Interestingly, during the data annotation process, it was noted that infrequently synthetic DNA strands were considerably longer than others, and it was decided to try including these as a separate ‘triple labeled long’ class. Although the purpose of this network is not to characterize molecule length (which is handled by subsequent post-processing), it was viewed as a potentially convenient way to further filter input datasets. These molecules were very rare however, and only 36 instances were found across the entire 1400 image dataset (roughly 5000 molecules). An annotated example image featuring one of these longer molecules is shown in figure 2(b).

The object detection network here is based on the YOLOv8 architecture [11], specifically the ‘small’ or ‘YOLOv8s’ variant of it. This size variant has 168 layers and 11.1 million parameters. Training/validation split was set to 80/20 and all other parameters were left on the ultralytics library documentation’s recommended settings. An in-depth description of the architecture, the loss function, and some of its predecessors [27–30] can be found in the recent paper by Wallace *et al* [26]. The optimizer used was AdamW, a modification to the Adam algorithm developed by Loshchilov and Hutter in 2019 [31]. On input during training, all images were resized to 640 × 640 pixels, and randomized mosaicking [32] and random left-to-right flipping were implemented for augmentation. To help with training convergence, we used transfer learning on the available pretrained versions of YOLOv8, which were trained on the Microsoft COCO dataset [33]. Optimal training time for the network was found to be around 60 epochs (an epoch is one full pass through the training data during the gradient descent process).

Object detection networks aim to predict both the locations and the classes of objects, thus the architecture must be designed carefully and the loss function must be more complex than binary cross entropy. Here we provide a basic summary of relevant components, however full details can be found discussed in the recent paper by Wallace *et al* [26]. The loss function for YOLOv8 is split into three main parts, the *bounding box loss*, the *class loss*, and the *generalized focal loss*. The bounding box loss measures the model’s ability to propose geometrically correct bounding boxes, and is a ‘Complete’ intersection over union loss, as detailed in Zheng *et al* [34]. The class loss is a multi-class cross entropy loss function that has been modified to allow for each bounding box separately to have probabilities corresponding to more than one class. The generalized focal loss, developed by Li *et al* in 2020 [35], is an additional function that works together with the class loss to speed up training by placing greater emphasis on training examples with which the model has difficulty, and most importantly allows for training on datasets that are extremely imbalanced. This is critical to the success of our application, because the vast majority of images will contain no objects of interest. The few images that do contain triple labeled DNA will typically only have one target strand out of potentially 10 or 20 in that frame.

## 3. Results and discussion

### 3.1. Image quality algorithm results

**Figure 3.**
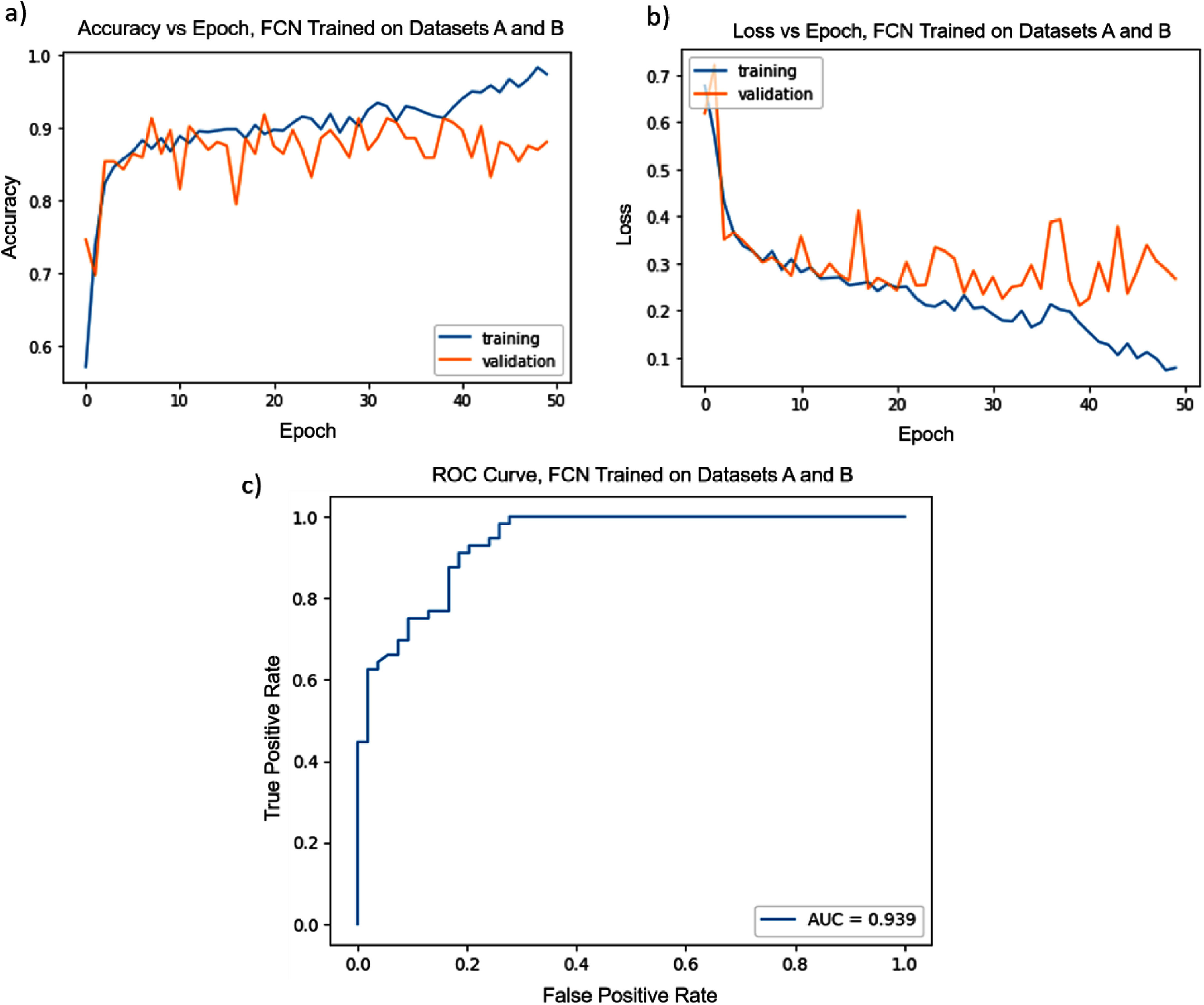
Training curve (a), loss curve (b), and ROC curve (c) for the best-tuned FCN, trained on both datasets A and B, with data augmentation and all other optimizations. The accuracy on the test Dataset C came out to 90% with AUC of 0.939.

The relative performance of the three image quality metrics is presented in table 1. For the FCN, combining Datasets A and B together for training produced the best results, with a ROC-AUC score of 0.939, and a raw accuracy score of 90% on the test Dataset C, shown in figure 3. It is worth noting that in between training on A and B separately and combining them, hyperparameters were further optimized and data augmentation was employed, and thus the large difference in final performance can at least partly be attributed to this. For reference, all datasets had approximately a 50/50 distribution of positive and negative classes. The final, best-case ROC-AUC score indicates a strong ability to reproduce the choices of AFM operators, and incorporating further test data from other users would likely strengthen this performance while increasing the network’s ability to generalize.

**Table 1.** Summary of best ROC-AUC and accuracy scores for the three selected methods of evaluating AFM images.

Method:	Fully convolutional network	Modified Laplacian of Gaussian	FFT-filtering
Accuracy:	96%	66.5%	60.0%
Test accuracy:	90.1%	N/A	N/A
ROC-AUC:	0.990	0.691	0.560

**Figure 4.**
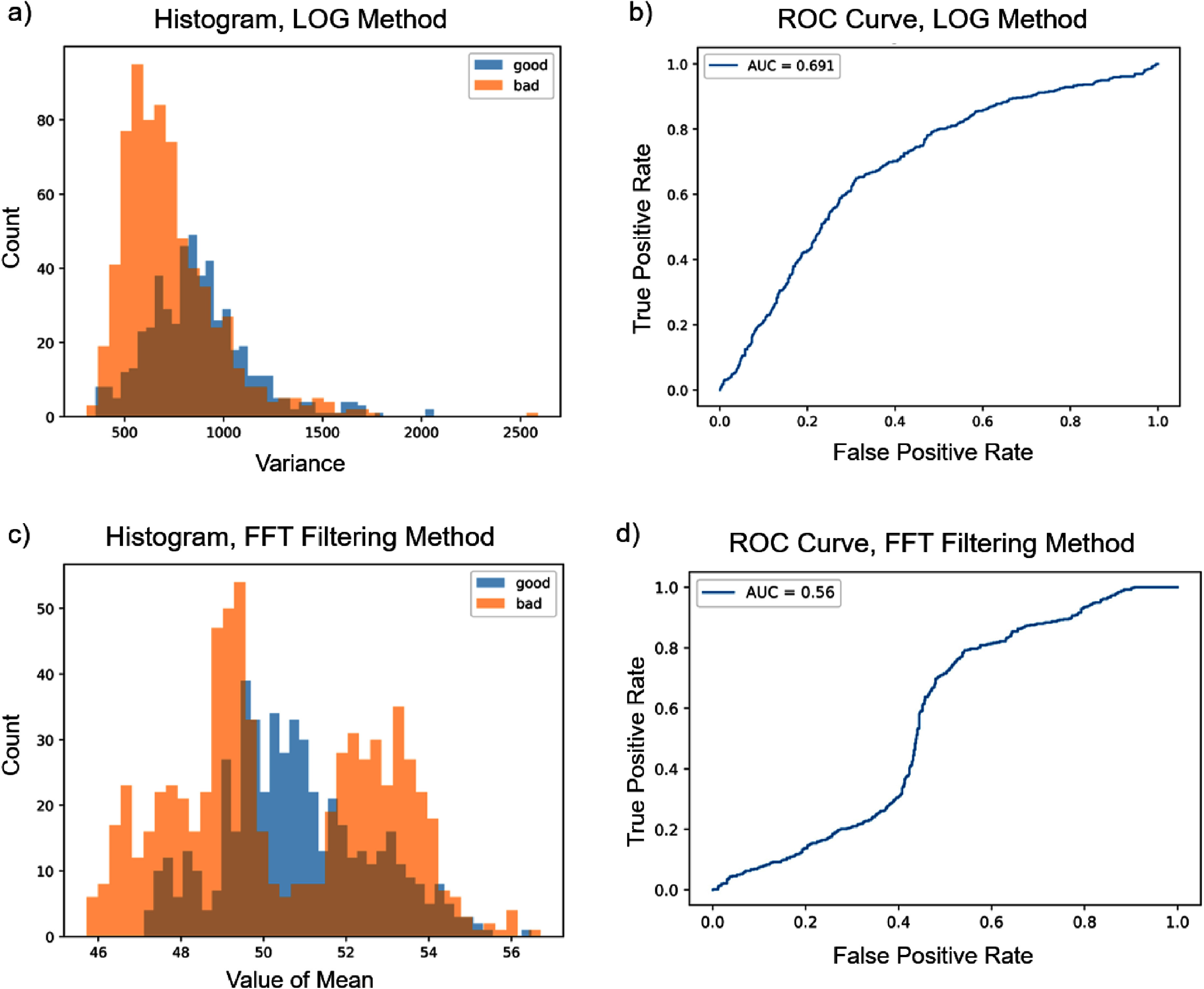
(a) and (c) Histograms of calculated mean values using the FFT-filtering method for blur detection at the optimal filter size of 40 × 40. X-axis is the mean of pixel intensity. (b) and (d) Corresponding ROC curves of the LOG method and FFT Filtering method respectively.

The LOG method proved to be less favorable. Figures 4(a) and (b) show a histogram and the ROC curve for the best optimized version of this blur detection method, with ROC-AUC score of 0.691. An important added step to this method which differs from existing literature, and which increased overall performance, was the addition of a segmentation scheme based on thresholding, binarization, and contour tracing (also implemented using OpenCV). This thresholding algorithm was used to place bounding boxes around the DNA strands to create small sub-images. The LOG method was then used only on the sub-images, effectively removing a large portion of the empty mica background from the LOG step. This removes some of the noise that is embedded in the background, and successfully increased the ROC-AUC of this method from 0.535 to 0.691. However, even after all optimizations, this method did not reproduce the classifications of the test set to a usable degree.

Figures 4(c) and (d) show the histogram and ROC curve for the best optimized version of the FFT filtering method. The only parameter to optimize in this case was the size of the filter (in frequency space), which was calculated via an exhaustive method and found to be 40 × 40 pixels. Recall that in this case, the metric for deciding whether an image is blurry or sharp is to calculate the mean pixel intensity after filtering the spatial frequencies of the image. It can clearly be seen that the histograms of both methods have significant overlap between the two classes, and as such there does not exist a single threshold that can achieve strong separation of the classes.

The main reason for poor performance of the FFT and LOG methods is the fact that the HSAFM images used in this scheme have a generally low signal-to-noise ratio. In the FFT calculation, high frequency noise in the mica background and the fact that the cantilever pressure blurring effect only affects the DNA strands hinders the filter’s ability to effectively separate the classes. As for the LOG method, the streaking and ‘shot’ noise (isolated regions of very high pixel intensity) cause problems with the second derivative calculation, even with Gaussian blurring to reduce their effect. A median filter was also employed as an alternative to reduce the effect of noise, with no significant performance change compared to Gaussian blurring. The typical use case for these two methods involves digital camera images, which use CMOS or CCD detectors, with superior signal-to-noise ratios. As a way to check that the FFT method was implemented in a reasonable way, this algorithm was benchmarked against a pre-labeled Kaggle ‘blurry image dataset’ [36].

### 3.2. Object detection algorithm results

**Figure 5.**
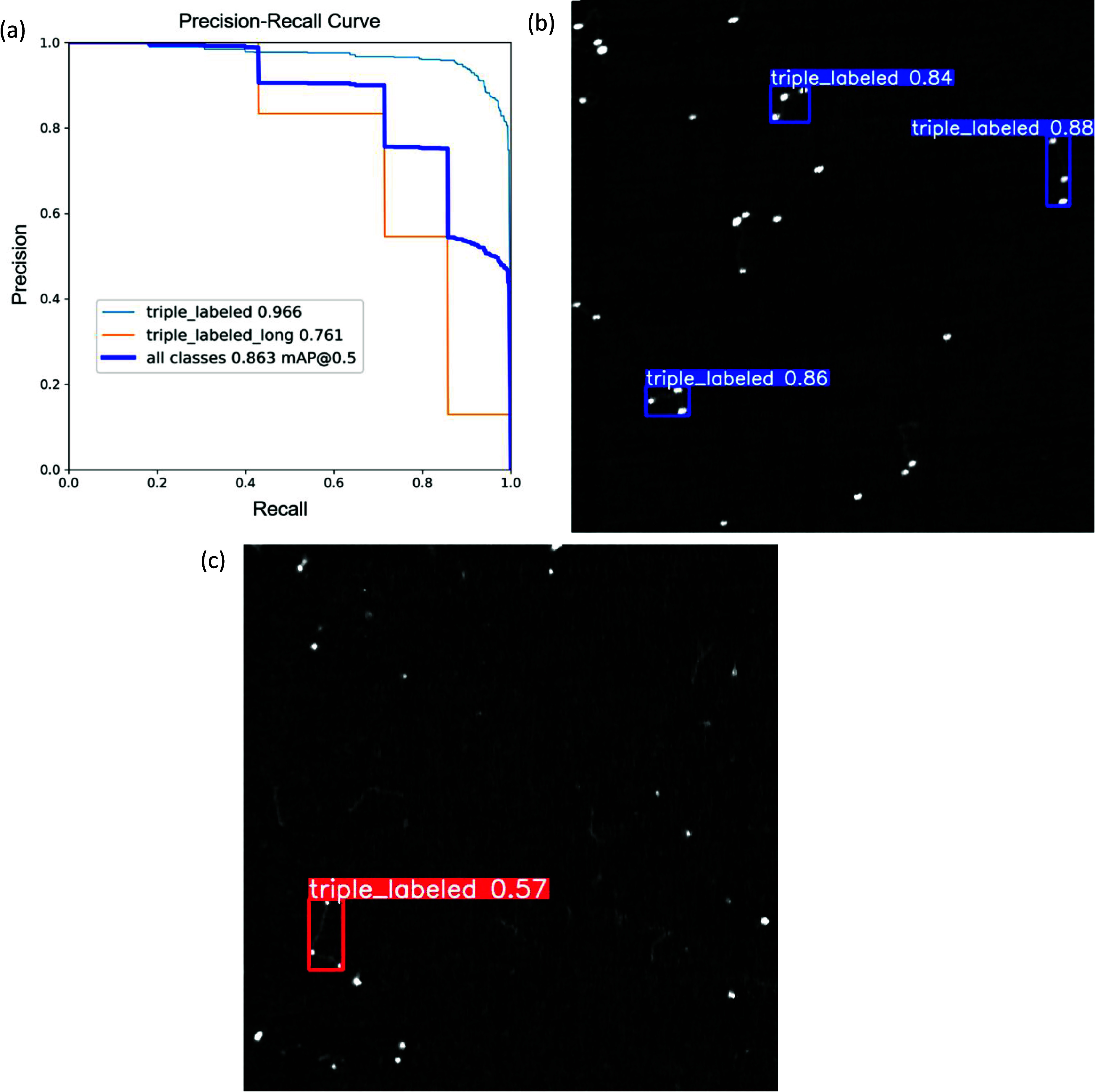
(a) Precision–recall curve for the object detection network, showing the mAP50 score of 0.863 (dark blue). The average precision scores for the separate classes were 0.966 (light blue) for the ‘triple labeled’ class and 0.761 (orange) for the ‘triple labeled long’ class. (b) Predictions made by the network on labeled validation DNA (blue bounding boxes). (c) An example image from the testing with cell line DNA, dataset for the object detection network, featuring one instance of the ‘triple labeled’ class (red bounding box). For (b) and (c), the numbers next to the labels represent the confidence of the network, or the estimated probability of the object being a triple labeled strand. Scale: image size is 2 × 2 *µ*m.

Loss, precision, and recall vs epoch curves for the training process of the model can be seen in figures 5, S4 and S5. Here, with a highly imbalanced dataset and multiple classes, precision, recall, and mean average precision (mAP50) are the appropriate metrics for evaluating model performance. Precision is the fraction of true positives out of all predicted positives (including false positives), while recall is the fraction of true positives out of all positives (including false negatives). mAP50 is the area under the precision–recall curve at a confidence threshold of 0.5, averaged across the two classes, while AP represents the area under the curve for a particular class. The network performed very well on the ‘triple labeled’ class (0.966 AP score), but not as well on the ‘triple labeled long’ class (0.761 AP), likely due to lack of training examples—only 36 instances were found in total. The synthetic molecule validation dataset ended up with only 7 examples of the long class and the synthetic molecule training set with 29.

To test the network, DNA from human cell lines as were prepared and around 20 000 images collected, with an unknown number of triple-labeled target molecules. After imaging was complete, the dataset was given to a trained AFM operator in our lab, who attempted to visually, manually inspect this data to find any potential candidates for correctly triple labeled targets, and found only one molecule. This process took an estimated total of 10 h. The object detection network identified 248 potential candidates, which were then directly inspected to find that 33 of them were real target molecules, based on the spacing of the Cas9 labels. Inference time was around 5–10 ms per image and the network was able to filter through the entire 20 000 image dataset in about three minutes, with only a few additional minutes of manual inspection required.

Figure 5(b) shows an example validation prediction from the synthetic molecule dataset. Figure 5(c) shows a test prediction of a triple-labeled molecule detected in the FMR1-enriched cell line. Figure 5(b) shows an annotated example image, featuring one of these ‘triple labeled long’ molecules in green, along with one of the ‘triple labeled’ molecules in red. A confusion matrix for the optimized algorithm and visualized example predictions made on the validation dataset can be found in figures S5 and S6, respectively.

## 4. Conclusions

**Image quality metrics.** The FCN algorithm showed strong ability to reproduce human-labeled results with 0.939 AUC, enabling the real-time automatic classification of HSAFM images of DNA, and producing a fractional metric of their overall quality. Using multiple AFM operators to contribute to the data labeling process afforded greater diversity in the dataset, likely leading to a more generalizable network overall. An interesting further experiment could be to have the same operators classify one another’s assigned datasets, allowing to quantify their differences of opinion. A scheme to incorporate all these datasets into the training and validation process could serve as an alternative form of data augmentation: for instance, by randomly selecting one of the three operators’ classifications to include, for each image as it is fed to the network during a training iteration. The training process could further be augmented by manually evaluating which testing examples the network is having difficulty with, and then intentionally collecting and labeling additional data of that type, adding it to the training dataset. This cyclic process is commonly called ‘semi-supervised learning’ and is a great fit in this scenario, since the diagnostic and sample prep processes are being actively improved.

The FCN runs quickly, and can be implemented to work in real-time with the HSAFM. Running this system in real-time can improve assay efficacy, since the process can be terminated early if the statistical confidence threshold is reached, and allow an HSAFM operator to experience real time feedback of sample quality. It is plausible that the adjustment of HSAFM parameters during scanning that is currently done manually by operators could be partly automated using feedback from the results of these algorithms, in a scheme analogous to that recently demonstrated by Krull *et al* on a scanning tunneling microscope [37].

**Object detection.** Generally, the network was more well-behaved with respect to recall vs precision, which aligns with the goals of this project. There exists a fundamental trade-off between recall and precision, and for this application we prefer a network that emphasizes recall. The application of this network to batches of HSAFM images is purely a way to filter out thousands of unusable frames that do not contain target molecules. Thus, the algorithm is best run with a low confidence threshold, approximately 0.25. This ensures that any strands that could be target strands are included, even if that means including some strands that are not true targets. This provides a trade-off where false negatives are more detrimental in this model than false positives, due to the rarity of true positives. The practical goal here is to sift through tens of thousands of images with the machine learning algorithm, then manually search the resulting few hundred images looking for the true target strands, which will then be characterized for their lengths separately using other image processing.

The chemical preparation process for DNA samples is additionally being improved, and will result in higher target concentrations in samples. This will, in turn, result in fewer total images required and positively affect the speed of diagnosis. Optimization of chemistry will also result in better signal to noise ratio for images and translate into measurable performance benefits for the object detection network. Another avenue of exploration is the addition of more classes to the object detection network. In principle, it is possible to have multiple targets all deposited on the same substrate, for example with label patterns of three, four, and five markers. These three different targets would correspond to three different mutations and could all be imaged in parallel. The object detection network would then distinguish between the three patterns and label them automatically for easier processing. The current network, optimized for the pattern of three, could be used or alternatively the training process could be started over again, using the pre-trained YOLOv8 architecture and parameters.

In the broader landscape of AFM data analysis, machine learning is increasingly being utilized to improve the speed, accuracy, and reproducibility of AFM techniques. Chiriboga *et al* developed a deep learning pipeline based on the YOLOv5 model for the identification of DNA origami triangles, breadboards, and tubes in AFM images [38]. The model was trained to detect DNA origami triangles and breadboards, using tubes as a control, and achieved a precision of 0.986 and 0.940, respectively [38]. This approach enabled rapid, high-throughput classification of nanostructures while reducing the need for manual analysis, demonstrating that machine learning can effectively distinguish between intended DNA assemblies and structural contaminants.

Beyond object detection, machine learning models also show promise in inferring molecular arrangements and enhancing low-quality AFM data. Priante *et al* presented a model capable of determining the atomic scale structure of monolayer and bilayer ice clusters on copper and gold substrates [39]. Their model could efficiently automate predictions of molecular arrangements, hydrogen bond arrangements, and substrate relaxation without the need for traditional, tedious manual calculations [39]. This capacity to resolve and classify complex molecular structures with atomic resolution demonstrates a broader potential for machine learning in molecular detection and structural inference.

## Data Availability

All data that support the findings of this study are included within the article (and any supplementary files).
